# Solubility of vitamin A in supercritical CO_2_: experimental study and thermodynamic modeling

**DOI:** 10.1038/s41598-021-92374-x

**Published:** 2021-08-05

**Authors:** Gowhar Ahmad Naikoo, Mohammad Saeedi Zadegan, Mona Zamani Pedram, Israr Ul Hassan, Waqar Ahmed, Golshan Yousefi Attar

**Affiliations:** 1 Department of Mathematics and Sciences, College of Arts and Applied Sciences, Dhofar University, Salalah, 211 Sultanate of Oman; 2 Faculty of Mechanical Engineering-Energy Division, K.N. Toosi University of Technology, No. 15-19, Pardis St., Mollasadra Ave., Vanak Sq., P.O. Box 19395-1999, 1999 143344 Tehran, Iran; 3 College of Engineering, Dhofar University, Salalah, 211 Sultanate of Oman; 4 School of Mathematics and Physics, College of Science, University of Lincoln, Lincoln, LN6 7TS UK; 5 Department of Chemical engineering, Islamic Azad University south Tehran Branch, Tehran, Iran

**Keywords:** Chemistry, Engineering

## Abstract

One of the best methods of extracting Vitamin A, as a helper of the immune body system and vision, was measured in supercritical carbon dioxide (SC-CO_2_); Mole fractions were gained at practical conditions in which the temperature was in the range of 303–323 K and the pressure range was 90–235 bar, respectively. Moreover, four Equation of States [Soave–Redlich–Kwong, Peng–Robinson, Stryjek–Vera and Dashtizadeh–Pazuki–Taghikhani–Ghotbi (DPTG)] were compared with the experimental data. Also, the mixing rules of Van der Waals (vdW1 and vdW2) selected to correlate the solubility data of vitamin A. The outcomes indicate that each of EOSs coupled with vdW2, as a method of estimating the physicochemical and critical properties, were correlated with the solubility data of vitamin A in SC-CO_2_ with more accuracy, in comparison with vdW1. Among the cubic EOSs, the DPTG model with vdW2 generated the most suitable correlation with the percentage average absolute relative deviation (Average Absolute Relative Deviation%) of 6.

## Introduction

Despite extensive research on the knowledge and importance of Vitamin A^1,2^ in the human body, the process of extracting and producing these materials from natural sources is not easy. Also, Vitamin A is soluble only in non-polar solvents due to having a bond O–H to the whole molecule (Table 1). In addition to this constraint, the operating conditions including temperature, pressure, exposure to air, light, moisture, water activity and pH are considerably affected by them which affects the characterization tests or equipment into practice which have to operate within permissible operating conditions^3^. Hence, separation and purification of natural vitamins can be identified as an optimum and economic step to produce pharmaceutical solids and reduce the side effects of synthetic ones^4^. Fortunately, extraction with supercritical fluid is a helpful method for purification of sensitive medicines from natural sources.

**Table 1:** Molecular structure of vitamin A^2^.

Name compound	Chemical formula	Structure	MW (g/mol)	$\lambda _{{max}}$(nm)	CAS number	Minimum purity
Retinol	C_20_H_3_O		286.45	325	68-26-8	95%

In fact, supercritical fluid extraction (SFE) is referred to a process in which one the extracting component is separated from another by means of fluids which are supercritical. Hence, these supercritical fluids were applied as the extracting solvent. In consequence they pave the way to mildly and selectively isolate substances from natural material^4–6^. This technology contributed to achieve extracts which are superior from a quality point of view with higher yield. Notably, there is no residual solvent. The SFE technique holds various features such as higher extraction efficiencies, simple and easy separation technology, no need for solvent recovery equipment and completion near to standard room temperature conditions^7^.

In this way, one of the best solvents amid them is SC-CO_2_ that is obtainable at high purities and low cost with low critical temperature and pressure (TC = 304.18 K and PC = 73.8 bar) and small surface tension as well as high selectivity^8^. Moreover, no residual solvent is detected by extract products taken from SC-CO_2_ Notably, this procedure is seductive to the food and beverage and medical industries. In comparison with other procedures, it brings about the least harm to the environment. CO_2_ has been labelled as the namely "green" solvent by the Federal Drug Administration (FDA), which is safe for industrial extractions^9^.

In this way, it is significant to determine the solubility of vitamins in supercritical phase (SCF). Such determination is suitable to design and develop the pharmaceutical processes; the computation and thermodynamic modeling should predict them^15^. Considerably, it is economically interesting to know the characteristic of phase equilibrium to optimize computation of separation processes.

One of the essential and significant approaches is modeling of solubility parameter in the SCF through EOS-based models with varied mixing rules, mathematically. This method is mainly employed to calculate phase equilibrium of neat fluids and mixtures as well as thermodynamic properties^10–12^. Also, to assess the solute solubility in supercritical fluid, the most pertinent models are the quadratic van der Waals equations of state as the most applicable models. These equations have been identified as the suitable ones which are able to estimate fluid phase equilibrium^13^.

Furthermore, a number of critical, physical and chemical properties are needed for several current models. It should be noted that such properties of solid hold critical sublimation pressure, Pitzer acentric feature and critical properties although they have never been achieved from an experimental point of view. Indeed, the consequential errors for different models are achieved; arising from the estimation method of the critical, physical, and chemical properties^14^.

The strengths and constraints of the employed equations of states are the two important factors which are able to develop a desirable mathematical model. As a result, this model is employed to estimate the solubility parameter in supercritical fluids.

Afterward, it is essential to precisely evaluate the use of reliable mixing rules which can predict the phase equilibrium. As a result, several researchers have confirmed the two-parameter van der Waals mixing rule displays the suitable ability to conclude the behavior of phase equilibria in nonpolar compounds or the ones with low polarity^15,16^. Recently, to predict the behavior of high polar materials with inherent deviation from the ideal condition, new mixing rules have been propounded.

The aforementioned equations (including SRK^17^, PR^18^, SV^19^ and DPTG^20^ with two adjustable parameters are performed in order to correlate the experimental data) with the solubility parameter can be measured and evaluated the predicted results with respect to the performed modification.

In addition, the EOSs selected in this work, have been chosen because of their special features which are completely attempted to the solubility extraction about vitamins. In other words, there are common properties between these equations include^17–20^:

- Ability to calculate to establish the relationship between temperature, pressure and volume of gases in supercritical situations.
- predicting gas-phase properties of solubility compounds, in vapor–liquid equilibria.
- In this EOSs, the effect of intermolecular forces and size of molecules are considered.

In order to evaluate its ability and estimate the deviation from the ideal state, the two-parameter mixing rules of van der Waals are practiced. The experimental data under the anticipated operating conditions for the binary vitamin A-SC-CO_2_ system was used.

The deviation of the calculated outcomes was calculated by the experimental data in order to evaluate the abilities of the selected models represented in terms of the AARD%. Moreover, the sublimation pressure is essential for the accuracy of the calculations which is anticipated to be used at the end of the calculations as a lever to validate the state equations.

In this work, a new study has been done for investigation the solubility points of vitamin A in supercritical solvent in two separate parts. In the first part (experimentally), the amount of molar fraction extracted by the setup designed (Setup description section) is recorded. In the second part (theoretically), these solubility points are compared with the calculated points by the EOS. Finally, total deviations between two numbers (experimental and calculations points) are investigated as a measure for select a more accurate EOS to predict the solubility points of vitamin A in the SC-CO_2_ solution.

## Methods

### Materials

Zagrosgas Company (Tehran, Iran) supplied the carbon dioxide (CO_2_) (CAS Number 124-38-9) at a least clarity of 99.99%. Retinol (Vitamin A) (CAS Number 68-26-8) with purity (GC) > 95% was obtained from Safirazma (Tehran, Iran). Also, analytical grade methanol (CAS Number 67-56-1) with purity of 99.8% was purchased from Safirazma (Tehran, Iran).

### Setup description

**Figure 1:**
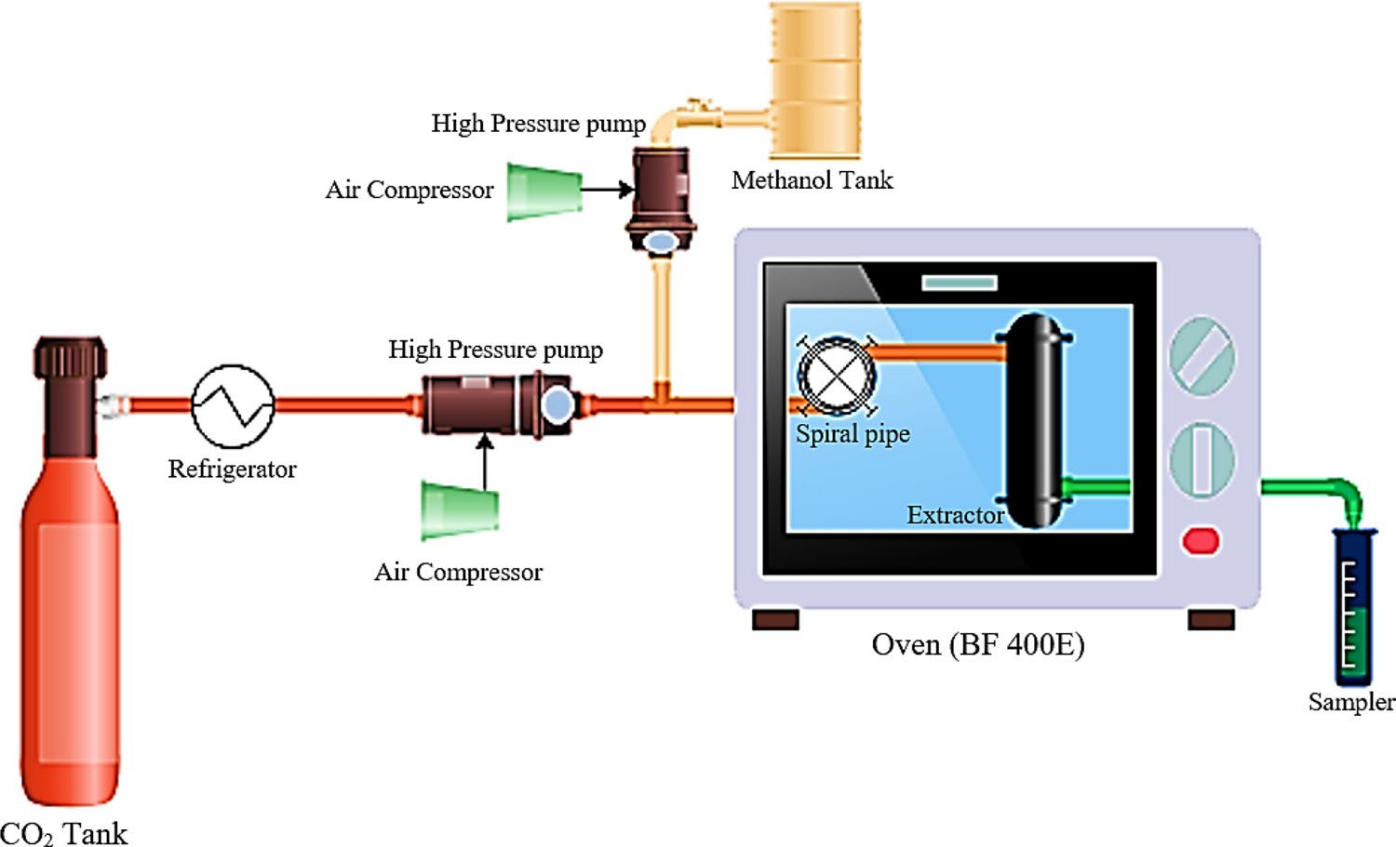
The setup shown schematic diagram of the experimental setup used for measuring solubility.

Figure 1 indicates the system used for measuring the equilibrium solubility. Before it was introduced into the fridge, the CO_2_ gas was passed through the pump by means of revealing the valve on a stainless-steel flask. Consequently, the gas was liquefied through dropping the temperature to − 20 °C so that it was prepared for pumping function by means of pressure controlling instruments (TEBCO, Iran) with an uncertainty of ± 0.1 MPa.

After CO_2_ pumped into the inlet solvent stream, the methanol tank (as a co-solvent in this section) and modulator valve controlled the solvent concentration. The inlet solvent concentration should be regulated. Thus, it is an adjustable factor as function of solute content based on simultaneous data from experimental setup. The pressure of the circuit was taken into the collection container through altering amount of modulator on the injection tap. This includes a certain volume of solvent (CO_2_ and methanol). Providing the thermal equilibrium condition with the inner part of the oven in shorter time, this equilibrium spiral pipe can be utilized for efficient mixing of CO_2_ and modulators. The cell was put inside carefully (FG Model, BF400E, Iran) to keep the operating temperature (with ± 0.5 K).

Roughly before the start of the extraction, 500 mg of powder Retinol were put into the static cell with the volume of 10 mL and due to the pressure drop, downward flowrate (in line with weight force of the materials) was used. Eluding physical entrainment of the unextracted Vitamin A (Rich Retinol), the tops of the pressurized equilibrium cell were connected by means of the stainless-steel disks before SC-CO_2_ was distributed into the cell, its pressure was enhanced up to the anticipated pressure before it is moved through the equilibrium cell at operational conditions. In the present research, 60 min was estimated to achieve the state of equilibrium (in the cell). It was shown to be sufficient through basic experiments.

In fact, the final sample (in the standard state) in two phases of gas (carbon dioxide) and liquid phase (solution containing methanol and vitamin A) It was stored in accumulation glass and entered the spectrophotometer. Finally, the solvent was used to clean the circuit.

As solubility values of Vitamin A were obtained in various conditions, they were noted by absorbency mensuration’s at ${{\uplambda }}_{{{\text{max}}}} {\text{~}}$ using a model 2100 UNICO UV–VIS spectrophotometer with 1000 nm Wavelength. The equilibrium mole fraction $~y_{A}$ and equilibrium solubility, $S(g/L)$ in SC-CO_2_ over the ranges of temperature and pressure obtained from below equation^21^:

1 $$y_{A} = n_{A} /\left( {n_{A} + n_{C} ~} \right)$$

where

2 $$n_{A} = \left( {C_{A} ~\left( {g/L} \right) \times V_{A} ~\left( L \right)} \right)/\left( {M_{A} ~\left( {{\text{g/mol}}} \right)~} \right)$$

3 $$n_{C} = \left( {V_{l} ~\left( L \right) \times \rho \left( {g/L} \right)} \right)/\left( {M_{C} ~\left( {{\text{g/mol}}} \right)~} \right)$$

where ${\text{n}}_{{\text{A}}} ~$ and ${\text{n}}_{{\text{C}}}$ are sample moles of vitamin A and solvent, ${\text{C}}_{{\text{A}}}$ is the concentration of vitamin A $\left( {g/L} \right)$ in the accumulation glass calculated by the curve of calibration. The volumes of the accumulation vial are denoted by ${\text{V}}_{{\text{A}}} \left( {\text{L}} \right)$ and ${\text{V}}_{{\text{l}}} \left( {\text{L}} \right)$ is the sampling circuit. Furthermore, ${\text{~~M}}_{{\text{A}}}$ and ${\text{M}}_{C}$ are the molecular weights of Vitamin A and CO_2_, respectively. By combining the aforementioned, the subsequent relation is achieved (Eq. (4)).

4 $${\text{y}}_{{\text{A}}} = \frac{{{\text{C}}_{{\text{A}}} \left( {\frac{{\text{g}}}{{\text{L}}}} \right) \times {\text{V}}_{{\text{S}}} \left( {\text{L}} \right) \times {\text{M}}_{{\text{C}}} \left( {\frac{{\text{g}}}{{{\text{mol}}}}} \right)}}{{{\text{C}}_{{\text{A}}} \left( {\frac{{\text{g}}}{{\text{L}}}} \right) \times {\text{V}}_{{\text{A}}} \left( {\text{L}} \right) \times {\text{M}}_{{\text{C}}} \left( {\frac{{\text{g}}}{{{\text{mol}}}}} \right) + {\text{V}}_{{\text{l}}} \left( {\text{L}} \right) \times {{\uprho }}\left( {\frac{{\text{g}}}{{\text{L}}}} \right) \times {\text{M}}_{{\text{A}}} \left( {\frac{{\text{g}}}{{{\text{mol}}}}} \right)}}$$

In order to obtain the $\left( {g/L} \right)$ of Vitamin A in SC-CO_2_ Eq. (4) was used^21^.

5 $$S\left( {g/L} \right) = \left( {C_{A} ~\left( {g/L} \right) \times V_{A} ~\left( L \right)} \right)/\left( {V_{l} ~\left( L \right)~} \right)$$

### Sublimation pressure estimation

Using supercritical solvents is considered as a new method in the purification process of pharmaceutical solids. When the solubility reliance on pressure and temperature is explicitly defined, it is possible to optimize the supercritical extraction. Notably, one of the significant design parameters is detected to be the sublimation pressure in the industrial solid–liquid separation processes.

In the present research, by means of the Clapeyron relation joined from the triple point pressure $P_{t}$ and temperature $T_{t}$ , the sublimation pressure ${\text{P}}^{s}$ at a temperature $T$ is assessed with assuming an insignificant functionality of the sublimation enthalpy to temperature^22^:

6 $$ln\left( {{\text{P}}^{s} /P_{t} ~} \right) = ~ - ~~\left( {{{\Delta }}H^{s} } \right)/R\left( {1/T - 1/T_{t} ~} \right)$$

In which, ${{\Delta }}H^{s}$ presents the sublimation enthalpy at the triple point which can be analyze by the equation below due the singularity of the triple point^23^.

7 $${{\Delta }}H^{s} = {{\Delta }}H^{v} + {{\Delta }}H^{f}$$

In which ${{\Delta }}H^{f} {\text{and}}$ ${{\Delta }}H^{v}$ are the fusion enthalpies and vaporization at the triple point, correspondingly. In many conditions, the triple point conditions are indefinite experimentally and the use of (Eqs. (1) and (2)) only needs some specific thermodynamic conditions. The method planned with this is established in two stages. Initially, it is supposed that the triple point temperature $T_{t}$ can be assessed by $DinT$ of the normal fusion temperature $T_{f}$. Certainly, the experimental values of transition temperatures in various literatures are scattered less than 0.1 K which is the difference between these two temperatures for the majority of heavy compounds^24^.

Based on this assumption,${{\Delta }}H^{f}$ in (Eq. (6)) can be projected by the synthesis enthalpy measured at the normal boiling point, thus application of Eq. (5) just needs specifying of Pt and ${{\Delta }}H^{v}$ at this temperature. In the next step, a correlation of vapor pressures is used to compute Pt and ${{\Delta }}H^{v}$ In this situation, experimental values of $P^{{sat}}$ are first interrelated through a relation due to temperature. In the current research, experimental statement of the vapor pressure applied^25^.

8 $$ln\left( {P^{{sat}} } \right) = A + B/\left( {T + C} \right) + DinT + ET~$$

In which bounds A, B, C, D, E were adjusted with the practical data. The equation of Yaws was used to obtain this statement in the case that very limited data points are available^26^.

Let subscript $C$ stand for the light (SC-CO_2_) component and let subscript $A$ stands for the heavy (Vitamin A) component. First, the general equation of equilibrium for Vitamin A at distinct operating conditions is written to calculate the solubility in the gas phase.

9 $$f_{A}^{s} = f_{A}^{{SCF}}$$

In which, superscript S characterizes the solid state. In this study, the supercritical fluid is presumed to not to be soluble in the solute stage. Additionally, the molar volume of the solute is pressure-independent and at sublimation point, the solute fugacity coefficient equals to 1. Solid phase is incompressible and pure. By considering the aforementioned assumptions, the derivation of equations is performed as following:

10 $$f_{A}^{s} = P_{A}^{{sub}} \phi _{A}^{{sub}} exp\left( {\mathop \smallint \limits_{{P_{A}^{{sub}} }}^{P} \frac{{V_{A}^{s} dP}}{{RT}}} \right)$$

where $P_{A}^{{sub}}$ is the sublimation pressure, $\phi _{A}^{{sub}}$ is the fugacity coefficient at sublimation pressure point, and $V_{A}^{s}$ is the solid molar volume, all at temperature $T$. For the fugacity of vapor-phase, we present fugacity coefficient $\phi _{{\text{A}}}^{{sub}}$ through eliciting its explanation.

11 $$f_{{\text{A}}}^{{SCF}} = Py_{{\text{A}}} \phi _{{\text{A}}}^{{SCF}}$$

We achieve the anticipated solubility of the heavy component in the gas phase by replacing and solving for $y_{A}$:

12 $$y_{A} = \left( {P_{A}^{{sub}} } \right)/P \times E$$

where

13 $$E = \frac{{\phi _{A}^{{sub}} {\text{exp}}\left( {\mathop \smallint \nolimits_{{P_{A}^{{sub}} }}^{P} \frac{{~V_{A}^{s} dP}}{{RT}}} \right)}}{{\phi _{A}^{{SCF}} }} = \frac{{\phi _{A}^{{sub}} exp\left( {\frac{{V_{A}^{s} \left( {P - P_{A}^{{sub}} } \right)}}{{RT}}} \right)}}{{\phi _{A}^{{SCF}} }}$$

The enhancement factor $E$ comprises three correction terms. The first term is the Poynting factor showing the effect of pressure on the pure solid fugacity which is considerable for the case of enhancement factor less than 2 or 3. The next correction, $\phi _{A}^{{sub}}$, considers non-ideality of the pure saturated vapor; the sublimation pressure of the solid is very small leading $\phi _{{\text{A}}}^{{sub}}$ to be almost equal to unity in most applied cases. The final term, $\phi _{A}^{{SCF}}$, is the last but the most significant. Nevertheless, $\phi _{{\text{A}}}^{{SCF}}$ is always far from unity and can yield great enhancement factors.

With the given numerical value of the $y_{A}$ parameter from previous experimental data, we can calculate the sublimation pressures at those conditions (Eq. (11)). Moreover, with this new data points about the sublimation pressure and the EOSs, we can predict new data points about solubility mole fractions ($y_{A}$).

In fact, this fulfills the fugacity equation (Eq. (8)) and it is essential to measure the solution to the fugacity equation to confirm the minimum Gibbs energy or since fugacity is needed for an adequate condition for a steady equilibrium^10^. As the temperatures, at which the solubility of Vitamin A was examined (303, 313 and 323 K), are well underneath its melting point, it is not necessary do a thermodynamic constancy analysis. Evidently, none of the considered temperatures are very close to the critical point of CO_2_ and they are amid the lower and upper critical end point.

To calculate $\phi _{A}^{{SCF}}$ we used different EOS-based models as described in later section. Consequently, we conclude an exclusive thermodynamically solution to the fugacity condition at each T and P allocating to the stable solid–fluid equilibrium^27^. The values for critical parameters, acentric factor, and sublimation pressure of Vitamin A should be put on Eq. (11) in order to calculate the solubility. Afterwards, we practice diverse EOS-based models for estimation of $\phi _{{\text{A}}}^{{SCF}}$.

### Equation of state-based models

According to the thermodynamic models mentioned in the previous sections, now they are examined in more detail. As already stated, the SRK^17^, PR^18^, SV^19^ and DPTG^20^ EOSs have been employed to calculate the fugacity constants in order to conclude the solubility of Vitamin A in SC-CO_2_ accompanied with relating vdW1 and vdW2 mixing rules (supplementary for more detail).

Holistically, cubic equations of state like (RK (and SRK), PR, SV and DPTG)^17–20^ are identified as the best choice modeling for phase equilibrium computations for multicomponent mixtures.

Moreover, it is required to conduct research so that the analytical cubic equations of state can be entirely developed. In this regard, improving the temperature dependency of attraction terms and the P(v) functional form so as to control the vapor pressure (estimation and enhance the prediction of volumetric properties are the main development of this study.

Based on perturbation theory, a new two-parameter cubic equation of state is suggested.

In the meantime, DPTG equation of state^20^ are used for the sake of precise calculation of the solubility factor since the outstanding role of solubility parameter has a significant consequence on the results of the model. Furthermore, the exactness of this EOS has been deliberated through estimation of the solubility in some supercritical fluids like CO_2_, methane and condensate gases, vaporization etc. Regarding previous studies^36^^,37^, the corresponding fluid phase behavior in both subcritical and supercritical fluid areas can be reliably foretold that the DTGP^20^ equations of state could indicated this.

### Compressibility fugacity

The fugacity coefficient of the system and component $i$ that shown in equations below, ($\emptyset ~\;{\text{and}}\;~\widehat{{\emptyset _{i} }}$ respectively) are identified as the two types of fugacity coefficients in thermodynamics^28,29^. In addition, $\ln \widehat{{\emptyset _{i} }}$ and $\ln \emptyset$ can be gained over the subsequent relation due to the functionality of fugacity coefficient and pressure:

14 $$\ln \widehat{{\emptyset _{i} }} = \frac{1}{{RT}}\mathop \smallint \limits_{0}^{P} \left( {\overline{{v_{i} }} - \overline{{v_{i}^{{ig}} }} } \right)dp = \mathop \smallint \limits_{0}^{P} \left( {\frac{{\overline{{v_{i} }} }}{{RT}} - \frac{1}{p}} \right)dp$$

15 $$\ln \emptyset = \frac{1}{{RT}}\mathop \smallint \limits_{0}^{P} \left( {v - v^{{ig}} } \right)dp = \mathop \smallint \limits_{0}^{P} \left( {\frac{{v_{t} }}{{nRT}} - \frac{1}{p}} \right)dp$$

Although the aforementioned approaches are really suitable in case the EOS for fluids is volume-explicit^30^, this is not the case for pressure-explicit EOS (where P is a function of T, $v$, and *x*_i_), such as the numerous cubic EOS and several non-cubic EOS. In such circumstances the independent P in Eqs. (14) and (15) should be distorted into $v$ and this can be understood by means of the relations specified in the supplementary for more details.

16 $$\ln \widehat{{\emptyset _{i} }} = - \frac{1}{{RT}}\mathop \smallint \limits_{\infty }^{{v_{c} }} \left( {\frac{{\partial P}}{{\partial n_{i} }}} \right)_{{T,v_{c} ,n_{{j\left( {j \ne i} \right)}} }} dv_{t} + \mathop \smallint \limits_{\infty }^{{v_{c} }} \frac{1}{{v_{c} }}dv_{c} - \ln z$$

17 $$\ln \emptyset = z - 1 - \ln z - \frac{1}{{RT}}\mathop \smallint \limits_{\infty }^{v} \left( {P - \frac{{RT}}{v}} \right)dv$$

Meanwhile furthermost EOS for fluids is pressure-explicit, Eq. (16) is more general than Eq. (14). Considering the component fugacity coefficients, most of the problems can be solved by the afore-said methods. Nonetheless, they bring about rough inadequacies. For instance, due to the intricated EOS in the first integral in Eq. (16) will be is problematic, or it cannot be recognized because the partial differentiation $\left( {\partial {\text{P}}/\partial n_{i} } \right)_{{{\text{T}},v_{c} ,n_{{j\left( {j \ne i} \right)}} }}$ which remarkably enhances the intricacy of integrated function. Necessarily, alternative approaches were taken in recent works^31^. Thus, they were easier than Eq. (16).

According to the previous studies $\ln \widehat{{\emptyset _{i} }}$ can be derived from $\ln \emptyset$ and also, A straight method for the derivation of $\ln \widehat{{\emptyset _{i} }}$ from $\ln \emptyset$ comes from the following relation^31^:

18 $$\ln \widehat{{\emptyset _{i} }} = \left[ {\frac{{\partial \left( {n\ln \emptyset } \right)}}{{\partial n_{i} }}} \right]_{{T,P,n_{j} }}$$

### Solution procedure

The optimal evaluation of the global value of the model binary interaction parameter is performed in terms of using the mixing rules of vdW1 and vdW2. The mixing rules comprise of adjustable parameters to consider exact chemical bonding forces such as hydrogen bonding and interaction due to the various size of mixture constituents. Depending on the mole fractions of mixture components, these mixing rules are employed in order to reach the slightest error when the predictions of the EOSs are associated with practical data. The aforementioned factors are recognized as binary interaction parameter which are used as a scale for assessing the deviation from the behavior anticipated in non-ideal systems. At a certain temperature the binary interaction parameter is achieved through regressing the model against experimental data of Vitamin A solubility.

**Figure 2:**
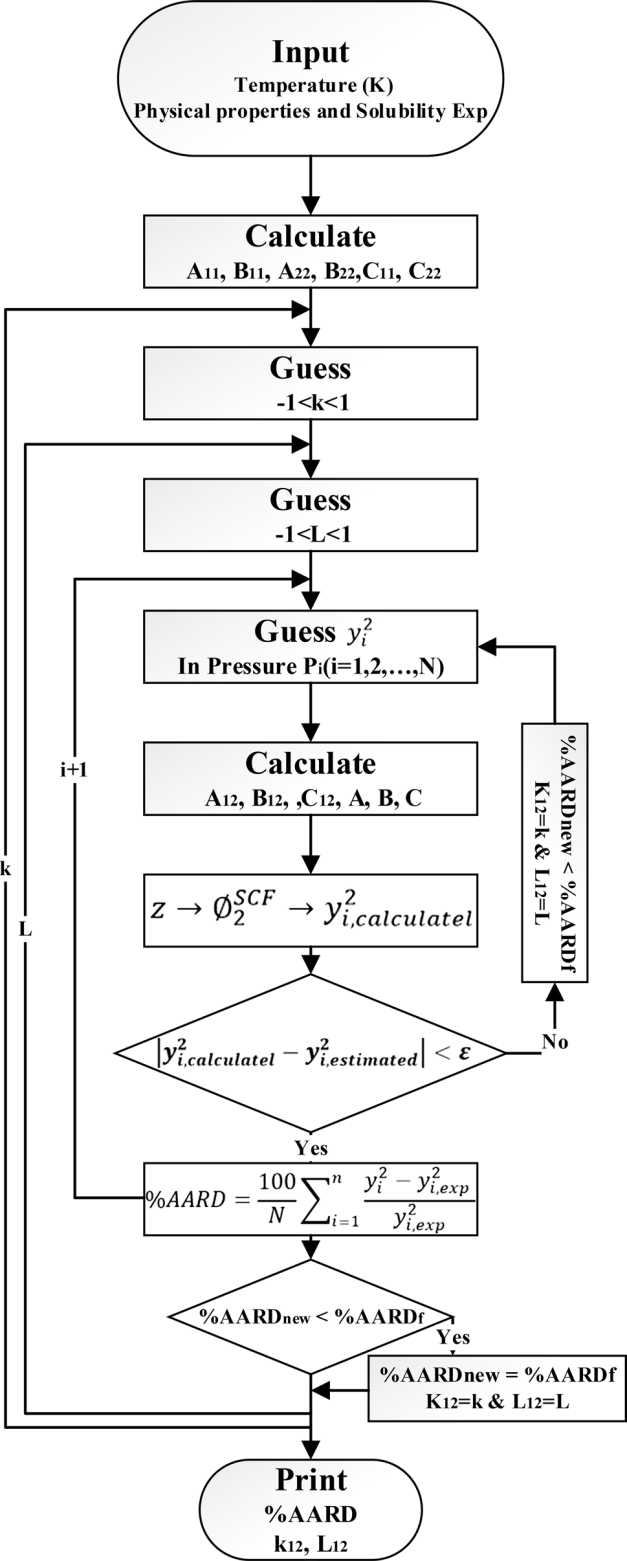
In the flowchart below, show the schematic of the calculations.

Figure 2 displays the stages needed so as to calculate the solubility. Furthermore, these stages indicate the several calculations done to calculate the solubility of Vitamin A in SC-CO_2_ at different operating conditions.

The compatible function is the AARD% between the calculated and experimental solubility. In which N is the number of experimental points at each temperature. The whole relations concluding the equations of state as well as mixing rules practiced in this research are presented in the supplementary for more detail.

19 $${\text{AARD}}\left( {{\% }} \right) = \frac{{100}}{N}\mathop \sum \limits_{{i = 1}}^{N} \frac{{\left| {y_{{i,c}} - y_{{i,e}} } \right|}}{{y_{{i,e}} }}$$

## Result and desiccation

### Experimental data

In this study when the pressure is in the range of 90–240 bar and the temperature range set at 303–323 K, equilibrium solubility data S (g/L) and the mole fraction (y) data of Vitamin A in SC-CO_2_ were specified.

Table 2 features the outcomes. It is worth noting that all experiments were repeated three times so that accurate and precise measurements was ensured. Remarkably, the comparative normal uncertainties were less than 6%. Furthermore, Fig. 5 divulges the uncertainty associated with the each of EOS. (The Span-Wagner equation^32^ was applied to contribute the density of SC-CO_2_. This equation is specifically formulated for CO_2_). In this regard, the mole fractions and solubility quantities of Vitamin A recorded varied from 2.18 × 10^–5^ to 1.964 × 10^–4^ and from 0.096 to 1.036, respectively. The maximum and minimum solubility of the Vitamin A were perceived at the temperature of 323 K and pressure of 235 bar and 303 K, 90 bar, correspondingly.

**Table 2:** Solubility of vitamin A in SC-CO_2_ at various temperatures and pressures.

Temperature (K)	Pressure (bar)	Mole fraction (× 10^3^)	Density (g/l)	Solubility ([g/l] × 10^3^)
303	90	0.0218	681.6	96.74
303	115	0.0292	758.1	144.1
303	130	0.0358	789	183.9
303	140	0.0412	806.5	216.2
303	155	0.0529	829.3	285.5
303	175	0.0601	855.3	334.5
303	185	0.0625	866.9	352.7
303	195	0.0721	877.7	411.8
303	220	0.0796	901.9	467.2
303	230	0.0819	910.7	485.4
303	245	0.0832	923	499.8
313	100	0.0168	561.3	61.36
313	120	0.0362	664.1	156.5
313	130	0.0541	696.5	245.3
313	140	0.0525	723.2	247.1
313	155	0.0663	756.3	326.3
313	170	0.0813	783.8	414.8
313	200	0.1255	828.3	676.6
313	235	0.138	869.1	780.8
313	245	0.1492	879.2	854.0
323	110	0.0268	462.2	80.62
323	135	0.0524	603	205.6
323	165	0. 1014	693	457.5
323	180	0.1402	725.3	661.9
323	195	0.1482	752.6	726.0
323	205	0.16	768.9	800.8
323	235	0.1964	810.5	1036

**Figure 3:**
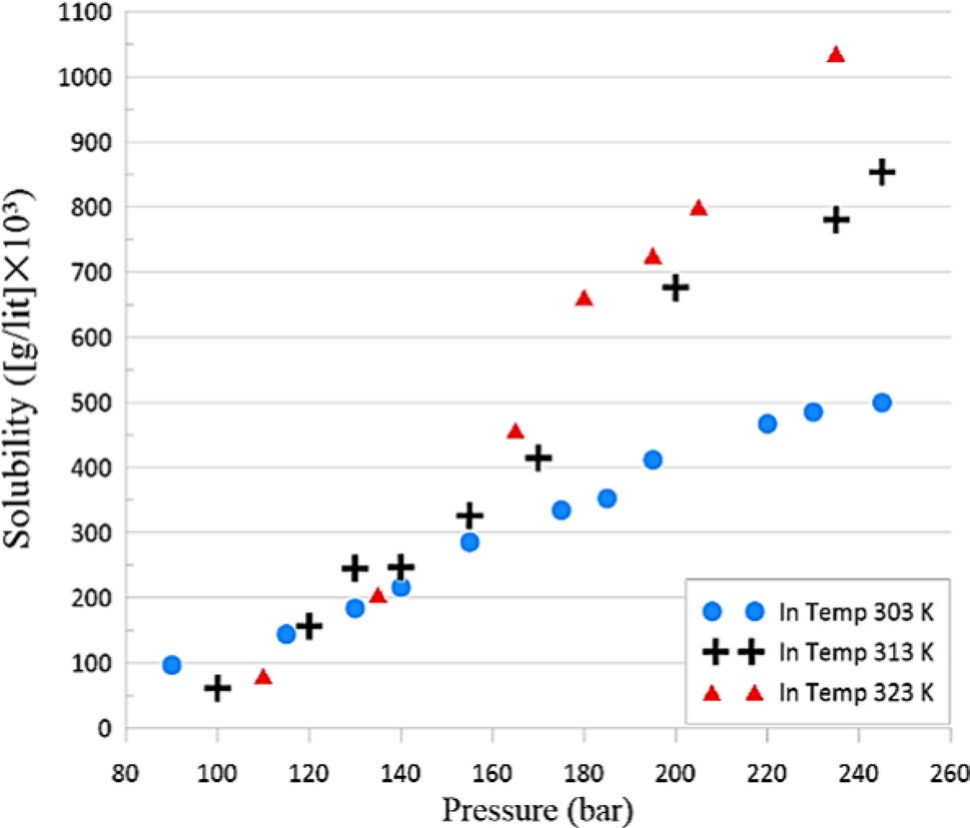
Solubility of Vitamin A vs pressure (at various temperatures).

According to Fig. 3, it can be deduced that solubility improves through increasing pressure at a constant temperature. This was brought about by the improved density along with the strong solvation property of SC-CO_2_ at higher pressures. A double effect on solubility is detected by the temperature in SC-CO_2_. This relies on in what way the solvent density and solute vapor pressure are well-adjusted^34^^,35^. Indeed, increasing the solute vapor pressure might arise from the enhancement of the solution temperature.

According to the Fig. 3, in pressure 110 bar, there is a cross-over pressure (at 303,313 and 323 K). This is despite the fact that in all cases, the solubility of vitamin A in SC-CO_2_ was over than 0.15 g/lit.

Henceforth, it affects stronger solvating power of SCF. Simultaneously, it is recognized that the SC-CO_2_ density might be diminished by a rise of temperature. This is detected to decline the total solvation property of the fluid.

Figure 3 indicates that the pressure range of 90–240 bar is in the beyond crossover pressure region for Vitamin A. Nonetheless, the density and solute vapor pressure are the leading factors at pressures under and beyond the crossover pressure region, correspondingly. For instance, the solubility may grow with temperature at pressures above the crossover pressure region. On the contrary to the 303 K which is recognized as the lowest one, the 323 K isotherm is highest isotherm. The solubility enhances through growing temperature for all pressures in the range examined. The behavior is influenced by two effects.

The primary is related with the increment of the solvating power of CO_2_ for the sake of density and the other one is the enhancement of solubility according to lessening the vapor pressure of Vitamin A. In this regards, similar results have been reported by researchers in clarification of the binary effect of temperature on the solubility in SC-CO_2_^33^.

### EOS-based models

As aforementioned, the EOS-based model was practiced in order to relate the solubility data of Vitamin A in SC-CO_2_. Furthermore, the consequences were associated in terms of AARD%. To calculate the Vitamin A solubility in SC-CO_2_, diverse combinations of EOSs such as the SRK^17^, the PR^18^, the SV^19^ and DPTG^20^ with two mixing rules (VdW1 and VdW2) are put into practice.

At three operation temperatures (303, 313 and 323 K) the PR^18^ and SRK^19^ EOSs with mixing rules of vdW1 and vdW2 contributed to the correlation results (Table 3). The optimum binary interaction factors ($k_{{ij}} ~$ and $l_{{ij}}$) as well as AARD% are held by them. The experimental data (27 data points) in extensive pressure at three levels of temperature were practiced to protect the generality of the current study.

**Table 3:** Correlation results for solubility of vitamin A in SC-CO_2_ solvent.

EOS	Parameters	In 303.15 K	In 313.15 K	In 323.15 K
SRK-vdW1	$kij$	0.07979	0.119786	0.167963
	$P_{{sub,2}}$	5.76E−06	0.000165	0.003333
	$AARD{\text{~}}\left( {{\% }} \right)$	7.54	11.58	9.35
SRK-vdW2	$kij$	0.632664	0.538128	0.602364
	$lij$	0.585396	0.513787	0.592264
	$P_{{sub,2}}$	0.267549	0.114635	0.993638
	$AARD{\text{~}}\left( {{\% }} \right)$	5.67	3.64	3.81
PR-vdW1	$kij$	0.080396	0.116453	1.170893
	$P_{{sub,2}}$	1.04E−05	2.41E−05	0.004242
	$AARD{\text{~}}\left( {{\% }} \right)$	6.96	10.70	8.68
PR-vdW2	$kij$	0.181497	0.517322	0.583477
	$lij$	0.115645	0.491264	0.571054
	$P_{{sub,2}}$	6.29E−05	0.102111	0.889204
	$AARD{\text{~}}\left( {{\% }} \right)$	6.36	3.62	3.82
SV-vdW1	$kij$	0.092516	0.127765	0.171397
	$P_{{sub,2}}$	1.045047	3.81E−05	0.004141
	$AARD{\text{~}}\left( {{\% }} \right)$	6.96	10.68	8.66
SV-vdW2	$kij$	0.625796	0.522877	0.546006
	$lij$	0.581154	0.490355	0.504091
	$P_{{sub,2}}$	0.266842	0.100798	0.562166
	$AARD{\text{~}}\left( {{\% }} \right)$	5.68	3.62	3.97
DPTG-vdW1	$kij$	0.353399	0.325725	0.34744
	$P_{{sub,2}}$	0.050702	0.106757	0.691951
	$AARD{\text{~}}\left( {{\% }} \right)$	5.78	4.52	4.09
DPTG-vdW2	$kij$	0.353399	0.391577	0.364307
	$lij$	0.252096	0.110898	0.034643
	$P_{{sub,2}}$	0.050702	0.21614	0.778508
	$AARD{\text{~}}\left( {{\% }} \right)$	5.78	3.65	4.04

**Figure 4:**
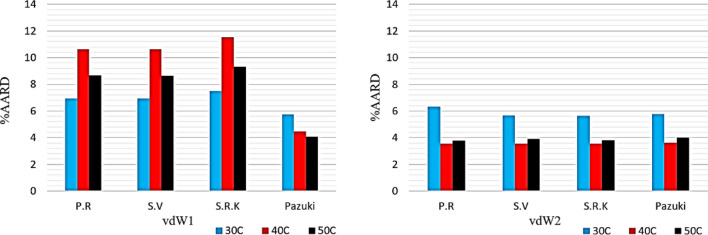
In the chart below shows the overall deviation of each of the models studied.

Figure 4 indicates that less average absolute relative errors (5.77% at 303 K, 4.51% at 313 K and 4.09% at 323 K) can be obtained from the combination of DPTG EOS^20^ along with applying vdW2 mixing rule. Definitely, it would be suitable for industrial uses. The deviations of the EOS-based models perhaps mostly owing to the point that cubic equations of state usually end to weak solubility predictions in supercritical fluid areas. In comparison with the results from other EOSs for this region, more reliable outcomes have been obtained from the DPTG EOS^20^.

According to Fig. 4, it is implied that the growth of solubility of such compound is made through the enhancement of pressure at constant temperature. The same influence is contributed by enhancing the temperature at fixed pressure.

**Figure 5:**
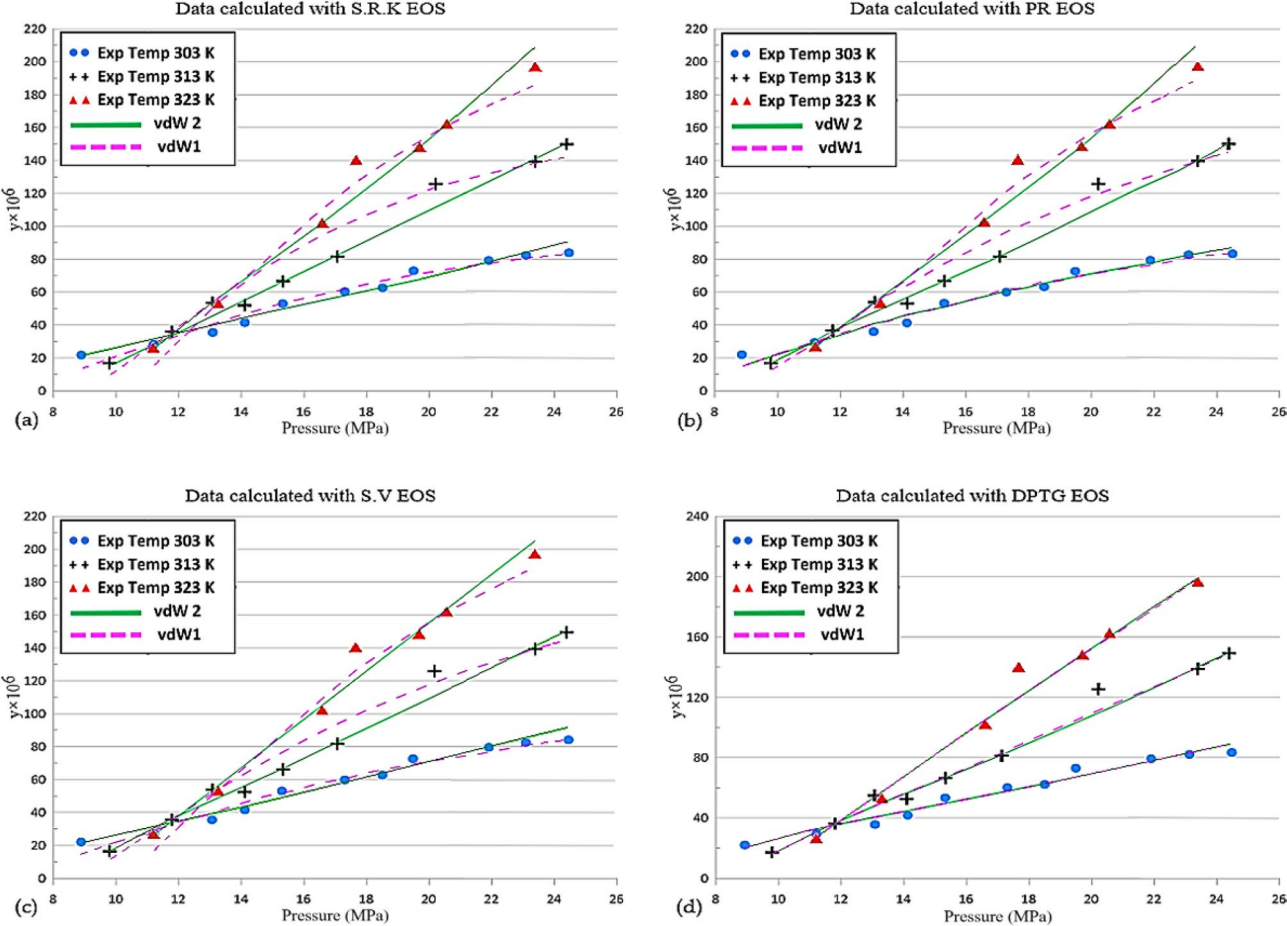
Comparison of experimental data (points) and calculated (line) solubility of vitamin A.

In such conditions, the solubility of Vitamin A is successfully represented by the chosen set of equations. Figure 5 illustrates the outcomes of AARD% according to the application of the (SRK, PR, SV and DPTG) EOSs^17–20^.

It is revealed that achievement of adjustable parameters is possible to estimate for wide pressure ranges. This figure likewise demonstrates that the assumption of the vdW2 mixing rules to gain consistent outcomes for the solubility of Vitamin A as a compromise has been obtained at a temperature range of 303–323 K for the parameters of the objective function.

Evidently, the two correlation parameters of $k_{{12}}$ and $l_{{12}}$ are properly extracted that the temperature reliance on the aforementioned parameters considered by means of the fitting procedure.

Holistically, DTPG EOS^20^ with vdW2 as mixing rule were capable of relating the solubility data of Vitamin A in SC-CO_2_ with the higher exactness in comparison with other EOSs.

The binary interaction parameters ($k_{{ij}}$ and $l_{{ij}}$) for the vdW1 and vdW2 mixing rules are linear functions of temperature as the following:

20 $$k_{{ij}} = A_{1} T + A_{2}$$

21 $$l_{{ij}} = A_{3} T + A_{4}$$

In which the coefficients $A$_1_, $A$_2_, $A$_3_,$~A_{4}$ were assessed through the analysis of linear regression (Table 4). These linear equations are appropriate to relate the interaction parameters for assessing the solubility of Vitamin A in SC-CO_2_ within the operating temperature range. Such linear equations are suitable to transmit the interface bounds for measuring the solubility of Vitamin A in SC-CO_2_ in the functioning temperature range. The consequences specified that the interaction parameter of $kij$ and $lij$ are linearly reliant on temperature in reverse.

**Table 4:** Adjustable parameters determined by linear regression fitting.

EOS	Interaction parameter			
	$kij$		$lij$	
	A	B	C	D
SRK-vdW1	0.0044	− 1.2581	–	–
SRK-vdW2	− 0.0015	1.0655	0.0003	0.4563
PR-vdW1	0.0545	− 16.619	–	–
PR-vdW2	0.0201	− 5.8666	0.0228	− 6.7379
SV-vdW1	0.0039	− 1.1045	–	–
SV-vdW2	− 0.004	1.8142	− 0.0039	1.7318
DPTGvdW1	− 0.0003	0.4355	–	–
DPTG-vdW2	0.0005	0.199	− 0.0109	3.5373

## Conclusion

Experimental measurement of fat-soluble Vitamin A in SC-CO2 from retinol (with 95% purification as a raw material) carried out from 303 to 323 K and at pressures from 90 to 235 bar. In this regard, solubility of Vitamin A (S (g/L)) and mole fractions (y) dissolved in SC-CO_2_ were in the variety of 0.096 to 1.036 and 2.18 × 10^–5^ to 1.964 × 10^–4^, respectively. The results were shown in Fig. 3, indicated the solubility dependency towards the variation of temperature and density. Also, with the density increases, the solubility of all substances increases at constant temperature. As long as the density remained constant, the solubility is increased resulting from the temperature increment although the improvement in the solid vapor pressure is caused by it. According to the results, the range of 0.7 g/kg was the one at which the solubility of fat-soluble vitamin A was measured in SC-CO_2_ under certain conditions. For fitting curves onto the solubility data of Vitamin A compound in SC-CO_2_, comparative studies were assumed so as to examine the employment of numerous cubic equations of state (SRK, PR, SV and DTPG)^17–20^ by various mixing rules (vdW1 and vdW2). Regressing the model against experimental solubility data was applied to find the optimized values of the model parameters. In the range of operating condition, 27 experimental data points were utilized for the thermodynamic equilibrium calculations.

As observed, applying DTPG EOS^20^ and VdW2 mixing rule directs to not as much complete average relative deviation (AARD 6%) of the outcomes from the consistent experimental values in comparison with others being capable of the higher accuracy in the Vitamin A solubility data in SC-CO_2_.

## Supplementary Information

Supplementary Information.

## List of symbols

a(T): Energy parameter of in the cubic EOS (Nm^4^ mol^−2^)
b: Parameter depend on Volume in cubic Equations-Of-State (m^3^ mol^−1^)
E: Solubility enhancement factor
EOS: Equation-Of-State
$k_{{ij}} , l_{{ij}}$: Mixing rule (binary interactions)
N: Experimental points
Ρ: Density (kg m^−^^3^)
P: Pressure (Pa)
R: Universal gas constant (J mol^−1^ K^−1^)
SRK: Soave–Redlich–Kwang
V: Molar volume (m^3^ mol^−1^)
vdW1: Van der Waals mixing rule with one adjustable parameter
vdW2: Van der Waals mixing rule with two adjustable parameters
y: Mole fraction solubility
k1: Pure compound adjustable parameters

## Greek symbols

α ($T_{r}$, ω): Function in the attractive parameter of the EOS’s
ω: Pitzer’s acentric factor

## Subscripts

1: Supercritical solvent
A: Vitamin A
C: Critical property
$$cal$$: Calculated mode
$$exp$$: Experimental mode
$$i$$, j: Components

## Superscripts

s: Solid (vitamin A)
sub: In sublimation
SCF: In supercritical phase
