# Supplementary material for: Solubility of vitamin A in supercritical CO2: experimental study and thermodynamic modeling
Source: Sci Rep. 2021 Aug 5;11:15917. doi: 10.1038/s41598-021-92374-x (PMC8342434; doi:10.1038/s41598-021-92374-x)
Supplement: Supplementary file 1 — Supplementary Information. [file 41598_2021_92374_MOESM1_ESM.docx]

**5. Supplementary**

**5.1. Appendix A**

In this work, Soave-Redlich-Kwong has been modified (Redlich-Kwong through substituting the term $a/{T^{0.5}}$ with a more common temperature reliant on term $a\left( T \right)$ [1]:

$P=RT/(v-b)-(a(T))/(v(v+b))$ (A1)

In analogy to the Soave-Redlich-Kwong equation, the parameters $a$ and *b* were calculated from critical point conditions [1]:

$a(T)=(0.42748R^{2} T_{c}^{2})/P_{c} \times\alpha(T_{r},\omega)$ (A2)

$\alpha\left( T,\omega\right)=\left[ 1+K\left( 1-\sqrt{T_{r}} \right) \right]^{2}$ (A3)

in which m represents the acentric factor as the following function:

$m=0.480+1.574\omega-0.26992\omega^{2}$ (A4)

The Peng-Robinson EOS is as below [2]:

$P=RT/(v-b)-(a(T))/(v(v+b)+b(v-b))$ (A5)

In analogy to the equation of Peng-Robinson, parameters $a$ and b were calculated from critical point conditions [2]:

$a(T)=(0.45724R^{2} T_{c}^{2})/P_{c} \times\alpha(T_{r},\omega)$ (A6)

$b=(0.07780RT_{c})/P_{c}$ (A7)

Dimensionless function of acentric factor and reduced temperature which is shown by $\alpha\left( T_{r},\omega\right)$ matches unanimity at the critical temperature:

$\alpha\left( T_{r},\omega\right)=\left[ 1+K\left( 1-\sqrt{T_{r}} \right) \right]^{2}$ (A8)

$K=0.37464+1.54226\omega-0.26992\omega^{2}$ (A9)

The original Stryjek–Vera EOS is [3]:

$P=RT/(v-b)-(a(T))/(v(v+b)+b(v-b))$ (A10)

$a(T)=(0.45724R^{2}T_{c}^{2})/P_{c} \alpha(T,\omega)$ (A11)

$b=(0.07780RT_{c})/P_{c}$ (A12)

And:

$\alpha\left( T,\omega\right)=\left[ 1+k\left( 1-\sqrt{T_{r}} \right) \right]^{2}$ (A13)

The expressions of $k$ in Equation(A19) are the main difference between the PRSV equation with the Peng-Robinson form which extended in the below relation [2].

$k=k_{0}+k_{1}\left( 1+\sqrt{T_{r}} \right)\left( 0.7-T_{r} \right)$ (A14)

Where, as in the PRSV equation $k_{0}$ is given by:

$k_{0}=0.378893+1.4897153\omega-0.171131848\omega^{2}+0.0196554\omega^{3}$ (A15)

The principal form of Dashtizadeh–Pazuki–Taghikhani–Ghotbi equation of state is shown as following [4]:

$P=RT/(v-b).(v+b)/v-a/v(v+b)$ (A16)

In analogy to the DPTG EOS, the parameters $a$ and *b* were calculated from critical point conditions:

$a(T)=0.47448 (R^{2} T_{c}^{2})/P_{c} \alpha(T_{r},\omega)$ (A17)

$b(T)=0.06824 (RT_{c})/P_{c}\beta(T_{r},\omega)$ (A18)

And:

$\alpha\left( T_{r} \right)=\left[ 1+m_{1}\left( 1-T_{r} \right)+m_{2}\left( 1-T_{r} \right)^{2}+m_{3}\left( 1-T_{r} \right)^{3} \right]^{2}$ (A19)

$m_{1}=0.2513+0.4178\omega-0.0207\omega^{2}$ (A20)

$m_{2}=-0.1382-0.5923\omega-1.0493\omega^{2}$  (A21)

$m_{3}=0.1931+1.3955\omega-0.1961\omega^{2}$ (A22)

$\beta\left( T_{r},\omega\right)=\left[ 1+n\left( 1-T_{r} \right) \right]$ (A23)

$n=0.2155-0.9069 \omega-0.206\omega^{2}$ (A24)

Mixing rules and combined laws [5]:

vdW0:

$A=\sum_{i}^{n} \sum_{j}^{n} y_{i}y_{j}A_{ij}=A_{11}y_{1}^{2}+A_{22}y_{2}^{2}+2A_{12}y_{1}y_{2}$ (A25)

$A_{12}=\sqrt{A_{1}A_{2}}$ (A26)

$B=\sum_{i}^{n} \sum_{j}^{n} y_{i}y_{j}B_{ij}=\sum_{i=1}^{2} y_{i}B_{ii}=B_{11}y_{1}+B_{22}y_{2}$ (A27)

$B_{12}=(B_{11}+B_{22} )/2$ (A28)

vdW1:

$A=\sum_{i}^{n} \sum_{j}^{n} y_{i}y_{j}A_{ij}=A_{11}y_{1}^{2}+A_{22}y_{2}^{2}+2A_{12}y_{1}y_{2}$ (A29)

$A_{12}=\sqrt{A_{1}A_{2}}\left( 1-k_{12} \right)$ (A30)

$B=\sum_{i}^{n} \sum_{j}^{n} y_{i}y_{j}B_{ij}=\sum_{i=1}^{2} y_{i}B_{ii}=B_{11}y_{1}+B_{22}y_{2}$ (A31)

$B_{12}=(B_{11}+B_{22} )/2$ (A32)

vdW2:

$A=\sum_{i}^{n} \sum_{j}^{n} y_{i}y_{j}A_{ij}=A_{11}y_{1}^{2}+A_{22}y_{2}^{2}+2A_{12}y_{1}y_{2}$ (A33)

$A_{12}=\sqrt{A_{1}A_{2}}\left( 1-k_{12} \right)$ (A34)

$B=\sum_{i}^{n} \sum_{j}^{n} y_{i}y_{j}B_{ij}=B_{11}y_{1}^{2}+B_{22}y_{2}^{2}+2B_{12}y_{1}y_{2}$ (A35)

$B_{12}=((B_{11}+B_{22} ))/2 (1-l_{12} )$ (A36)

**5.2. Appendix B:**

The differential Eq. (14) can be rewritten as:

$\bar{v}_{i}dP=\left( \frac{\partial v_{t}}{\partial n_{i}} \right)_{T,P,n_{j}} dP=-\left( \frac{\partial v_{t}}{\partial P} \right)_{T,n_{i},n_{j}}\left( \frac{\partial P}{\partial n_{i}} \right)_{T,v_{t},n_{j}}dP$ (B1)

In the case of thermodynamic closed system at certain temperature, it will be:

${dv}_{t}=\left( \frac{\partial v_{t}}{\partial P} \right)_{T,n_{i},n_{j}}dP$ (B2)

So, Eq.(B1) can be transformed into:

$\bar{v}_{i}dP=\left( \frac{\partial v_{t}}{\partial n_{i}} \right)_{T,P,n_{j}}dP=-\left( \frac{\partial P}{\partial n_{i}} \right)_{T,v_{t},n_{j}}dv_{t}$ (B3)

It will be as below for perfect gas mixture ($ig$)

$\bar{v}_{t}^{ig}dP=\left( \frac{\partial P}{\partial n_{i}} \right)_{T,v_{t}^{ig},n_{j}} dv_{t}^{ig}=-\frac{RT}{v_{t}^{ig}}dv_{t}^{ig}=-RTd\ln v_{t}^{ig}$ (B4)

Where $v_{t}^{ig}$ can be stated with $v_{t}$ and z as the total volume and compressibility factor of the real fluid, respectively:

$v_{t}^{ig}=nRT/P=nRT/(nzRT/v_{t} )=v_{t}/z$ (B5)

So, Eq. (A.4) can also be rewritten as:

$\bar{v}_{t}^{ig}dP=RTd\ln z-\frac{RT}{v_{t}}dv_{t}$ (B6)

With Eqs. (A3) and (A6), we can transform Eq (14) can be changed into Eq (16). Likewise, Eq (15) can be changed into Eq (17) by dint of the subsequent relation:

$vdP=d(Pv)-Pdv=RTdz-Pdv$ (B7)

$\frac{dP}{P}=d\ln P=d\ln\left( \frac{zRT}{v} \right)=d\ln z-d\ln v=d\ln z-\frac{1}{v}dv$ (B8)

The Fugacity Coefficient from Soave-Redlich-Kwong [1] is:

$\ln\phi_{2}^{SCF}=-\ln\left( \frac{P\left( \left( \frac{ZRT}{P} \right)-B \right)}{RT} \right)-\frac{-B_{22}+l_{12}\left( B_{11}+B_{22} \right)\left( 1-y_{2} \right)^{2}}{\left( \frac{ZRT}{P} \right)-B}-\frac{1}{RT}\left[ \frac{A}{B}.\frac{B_{22}-l_{12}\left( B_{11}+B_{22} \right)\left( 1-y_{2} \right)^{2}}{\left( \frac{ZRT}{P} \right)+B} \right]-\frac{1}{BRT}\ln\left( 1-\frac{B}{\left( \frac{ZRT}{P} \right)} \right)\times\left[ 2A_{22}y_{2}+2\sqrt{A_{11}A_{22}}\left( 1-k_{12} \right)\left( 1-y_{2} \right)-\frac{A}{B}\left( B_{22}-l_{12}(B_{11}+B_{22})\left( 1-y_{2} \right)^{2} \right) \right]$ (B9)

The Fugacity Coefficient from Peng-Robinson [2] is:

$\ln\phi_{2}^{SCF}=-\ln\left( \frac{P\left( \left( \frac{ZRT}{P} \right)-B \right)}{RT} \right)+\frac{Z-1}{B}\left[ B_{22}-l_{12}\left( B_{11}+B_{22} \right)\left( 1-y_{2} \right)^{2} \right]-\frac{1}{2\sqrt{2}RT}\ln\frac{v+B\left( 1+\sqrt{2} \right)}{v+B\left( 1-\sqrt{2} \right)}\times\left[ \frac{2A_{22}y_{2}+2\sqrt{A_{11}A_{22}}\left( 1-k_{12} \right)(1-y_{2})}{B}-\frac{A}{B^{2}}\left( B_{22}-l_{12}\left( B_{11}+B_{22} \right)\left( 1-y_{2} \right)^{2} \right) \right]$ (B10)

The Fugacity Coefficient from Stryjek-Vera [6] is:

$\ln\phi_{2}^{SCF}=-\ln\left( \frac{P\left( \left( \frac{ZRT}{P} \right)-B \right)}{RT} \right)+\frac{Z-1}{B}\left[ B_{22}-l_{12}\left( B_{11}+B_{22} \right)\left( 1-y_{2} \right)^{2} \right]-\frac{1}{2\sqrt{2}RT}\ln\frac{v+B\left( 1+\sqrt{2} \right)}{v+B\left( 1-\sqrt{2} \right)}\times\left[ \frac{2A_{22}y_{2}+2\sqrt{A_{11}A_{22}}\left( 1-k_{12} \right)(1-y_{2})}{B}-\frac{A}{B^{2}}\left( B_{22}-l_{12}\left( B_{11}+B_{22} \right)\left( 1-y_{2} \right)^{2} \right) \right]$ (B11)

The Fugacity Coefficient from Dashtizadeh–Pazuki–Taghikhani–Ghotbi [4] is:

$\ln\phi_{2}^{SCF}=-\ln Z-2\ln\left( 1-\frac{B}{\left( \frac{ZRT}{P} \right)} \right)-2\left( \frac{-B_{22}+l_{12}\left( B_{11}+B_{22} \right)\left( 1-y_{2} \right)^{2}}{\left( \frac{ZRT}{P} \right)-B} \right)-\frac{1}{RT}\left[ \frac{A}{B}.\frac{B_{22}-\left( B_{11}+B_{22} \right)l_{12}\left( 1-y_{2} \right)^{2}}{\left( \frac{ZRT}{P} \right)+B} \right]-\frac{1}{BRT}\ln\left( 1+\frac{B}{\left( \frac{ZRT}{P} \right)} \right)\times\left[ 2A_{22}y_{2}+2\sqrt{A_{11}A_{22}}\left( 1-k_{12} \right)\left( 1-y_{2} \right)-\frac{A}{B}\left( B_{22}-l_{12}\left( B_{11}+B_{22} \right)\left( 1-y_{2} \right)^{2} \right) \right]$ (B12)

Compressibility coefficient from Soave-Redlich-Kwong [1] is:

$Z^{3}-Z^{2}+\left[ \frac{AP}{\left( RT \right)^{2}}-\frac{\left( BP \right)^{2}}{\left( RT \right)^{2}}-\frac{BP}{RT} \right]Z-\left[ \frac{{ABP}^{2}}{\left( RT \right)^{3}} \right]=0$ (B13)

Compressibility coefficient from Peng-Robinson [2] is:

$0=Z^{3}-\left[ 1-\frac{BP}{RT} \right]Z^{2}+\left[ \frac{AP}{\left( RT \right)^{2}}-3\left( \frac{BP}{RT} \right)^{2}-\frac{2BP}{RT} \right]-\left[ \frac{{ABP}^{2}}{\left( RT \right)^{3}}-\left( \frac{BP}{RT} \right)^{2}-\left( \frac{BP}{RT} \right)^{3} \right]$ (B14)

Compressibility coefficient from Stryjek–Vera is:

$Z^{3}-\left[ 1-\frac{BP}{RT} \right]Z^{2}+\left[ \frac{AP}{\left( RT \right)^{2}}-3\left( \frac{BP}{RT} \right)^{2}-\frac{2BP}{RT} \right]-\left[ \frac{{ABP}^{2}}{\left( RT \right)^{3}}-\left( \frac{BP}{RT} \right)^{2}-\left( \frac{BP}{RT} \right)^{3} \right]=0$ (B15)

Compressibility coefficient from Dashtizadeh–Pazuki–Taghikhani–Ghotbi [4] is:

$Z^{3}-Z^{2}-\left[ \frac{\left( BP \right)^{2}}{\left( RT \right)^{2}}-\frac{AP}{\left( RT \right)^{2}}-\frac{2BP}{RT} \right]Z-\left[ \frac{{ABP}^{2}}{\left( RT \right)^{2}}+\frac{\left( BP \right)^{2}}{\left( RT \right)^{2}} \right]=0$ (B16)

**References**

[1] G. Soave, “Equilibrium constants from a modified Redlich-Kwong equation of state,” *Chem. Eng. Sci.*, vol. 27, no. 6, pp. 1197–1203, 1972. <https://doi.org/10.1016/0009-2509(72)80096-4>

[2] D. Y. Peng and D. B. Robinson, “Industrial Engineering Chemistry Fundamentals.” American Chemical Society, 1976.

[3] R. Stryjek and J. H. Vera, “PRSV: An improved Peng—Robinson equation of state for pure compounds and mixtures,” *Can. J. Chem. Eng.*, vol. 64, no. 2, pp. 323–333, 1986. <https://doi.org/10.1002/cjce.5450640224>

[4] A. Dashtizadeh, G. R. Pazuki, V. Taghikhani, and C. Ghotbi, “A new two-parameter cubic equation of state for predicting phase behavior of pure compounds and mixtures,” *Fluid Phase Equilib.*, vol. 242, no. 1, pp. 19–28, 2006. <https://doi.org/10.1016/j.fluid.2006.01.005>

[5] A. Z. Panagiotopoulos and R. C. Reid, “New mixing rule for cubic equations of state for highly polar, asymmetric systems,” 1986. 10.1021/bk-1986-0300.ch028

[6] R. Stryjek and J. H. Vera, “PRSV2: a cubic equation of state for accurate vapor—liquid equilibria calculations,” *Can. J. Chem. Eng.*, vol. 64, no. 5, pp. 820–826, 1986. <https://doi.org/10.1002/cjce.5450640516>
